# Physicochemical and antioxidant properties of Bangladeshi honeys stored for more than one year

**DOI:** 10.1186/1472-6882-12-177

**Published:** 2012-10-08

**Authors:** Asiful Islam, Ibrahim Khalil, Nazmul Islam, Mohammed Moniruzzaman, Abdul Mottalib, Siti Amrah Sulaiman, Siew Hua Gan

**Affiliations:** 1 Human Genome Centre, School of Medical Sciences, Universiti Sains Malaysia, 16150 Kubang Kerian, Kelantan, Malaysia; 2 Department of Pharmacology, School of Medical Sciences, Universiti Sains Malaysia, 16150 Kubang Kerian, Kelantan, Malaysia; 3 Department of Biochemistry and Molecular Biology, Jahangirnagar University, Savar, Dhaka, 1342, Bangladesh; 4 Laboratory Services Division, Bangladesh Institute of Research and Rehabilitation in Diabetes, Endocrine and Metabolic Disorders Hospital (BIRDEM), Shahbag, Dhaka, Bangladesh

**Keywords:** Bangladeshi honey, Antioxidant, Physicochemical, Phenolics, Proline

## Abstract

**Background:**

There is no available information on physicochemical and antioxidant properties on Bangladeshi honey. We investigated five different monofloral and three different multifloral honey samples collected from different parts of Bangladesh.

**Methods:**

The levels of phenolics, flavonoids, ascorbic acid, ascorbic acid equivalent antioxidant content (AEAC), proline, protein and antioxidants were determined in the honey samples using ferric reducing antioxidant power (FRAP) and 1,1-diphenyl-2-picrylhydrazyl (DPPH) assays.

**Results:**

The highest level of phenolic was 688.5 ± 5.9 mg Gallic acid/kg, and the highest level of flavonoid was 155 ± 6.9 mg Catechin/kg. The highest color intensity was 2034.00 ± 17.5 mAU, and the highest protein content was 8.6 ± 0.0mg/g. High levels of proline (2932.8 ± 3.7 mg/kg), ascorbic acid (154.3 ± 0.3 mg/kg), AEAC (34.1 ± 1.4mg/100 g) and FRAP (772.4 ± 2.5 μmol Fe (II)/100 g) were detected in some of the samples, especially the multifloral honey samples, indicating good antioxidant properties. A strong positive correlation was found between phenolics, flavonoids, DPPH, FRAP and color intensity, indicating that in addition to total phenolic and flavonoid concentrations, color intensity and amino acid are good indicators of the antioxidant potential of honey. Except for a single sample (BDH-6), the honey samples stored for 1.5 years at room temperature still had 5-hydroxymethylfurfural (HMF) values within the recommended range (mean = 10.93 mg/kg), indicating that the rate of HMF production in Bangladeshi honey samples is low.

**Conclusion:**

It is postulated that the low rate of HMF formation could be attributed to the acidic and low moisture content in the samples. In general, multifloral honeys have higher antioxidant properties based on their high levels of phenolics, flavonoids, AEAC, DPPH and FRAP when compared to monofloral honeys. We also found that monofloral honey samples from *Guizotia abyssinica* and *Nigella sativa* had high antioxidant properties.

## Background

Honey is a complex mixture of 82.0% carbohydrates (sucrose, fructose, maltose), 0.3% protein, 17.0% water and 0.7% minerals, vitamins and antioxidants [1]. Honey contains a variety of phytochemicals and other substances, such as organic acids, vitamins and enzymes, which may serve as a source for dietary antioxidants. The antioxidant capacity is probably the most important property of honey and is affected by the presence of flavonoids, phenolic acids, ascorbic acid, catalase, peroxidase, carotenoids and products of Maillard reactions [2]. High levels of flavonoids, phenolic acids, ascorbic acid, catalase, peroxidase and carotenoids ensure a high level of antioxidants in honey [3]. Honey consumption by humans has been reported to increase total plasma antioxidant and reducing capacity, which can be protective to human health.

The comparative physicochemical characterization of different honeys from other regions of the world has been extensively conducted, including Algeria [4], India [5], Slovenia [6] and Malaysia [7]. In Bangladesh, honey is produced and consumed on a large scale. Sundarbans, which is the largest mangrove forest in the world, consists of 334 plant species and is ideal for giant honey bees (*Apis dorsata*) and honey collectors. However, there is still a lack of information on the comparative physicochemical and biochemical properties of different types of Bangladeshi honeys.

Because honey samples collected from different floral sources and geographic locations may vary in terms of physicochemical properties and antioxidant capacity, especially in monofloral versus multifloral honey, we attempted to analyze eight different honey samples collected from different regions of the country. To date, no data is available on the physicochemical and antioxidant properties of honey samples from Bangladesh. Recently, honeys from Cuba have been reported to have the capacity to prevent or reduce oxidative damage of erythrocytes [8] and lipid peroxidation associated inflammatory diseases in which oxidative stress is involved [9]. Another current study has shown that Malaysian Gelam honey has anti-inflammatory effect by decreasing the immune response against inflammation [10] and by having protective effects against organ failure [11]. Honeys from different regions have been reported to have more potent antioxidant activities than the vitamins A, C and E, as well as to have the capacity to reduce oxidative stress related chronic or degenerative diseases [12]. The capacity of antioxidants has been investigated by various methods. However, due to the fact that some methods give inconsistent and conflicting results [13], we have selected several combination of assays such as ascorbic acid equivalent antioxidant content (AEAC), 1,1-diphenyl-2-picrylhydrazyl (DPPH) and ferric reducing antioxidant power (FRAP) to indicate antioxidant capacities. The objective of the current study was to investigate the physicochemical and antioxidant properties of Bangladeshi honeys of monofloral and multifloral origin.

## Methods

### Chemicals

Ascorbic acid; proline; DPPH; 2,4,6-tris(1-pyridyl)-1,3,5-triazine (TPTZ); 5-hydroxymethylfurfural (HMF) and Folin–Ciocalteu’s reagent were purchased from Sigma-Aldrich (St. Louis, Mo., U.S.A.). Sodium carbonate (Na_2_CO_3_), aluminum chloride (AlCl_3_), sodium nitrite (NaNO_2_) and sodium hydroxide (NaOH) were purchased from Merck (Darmstadt, Germany). All chemicals used were of analytic grade.

### Honey samples

**Figure 1:**
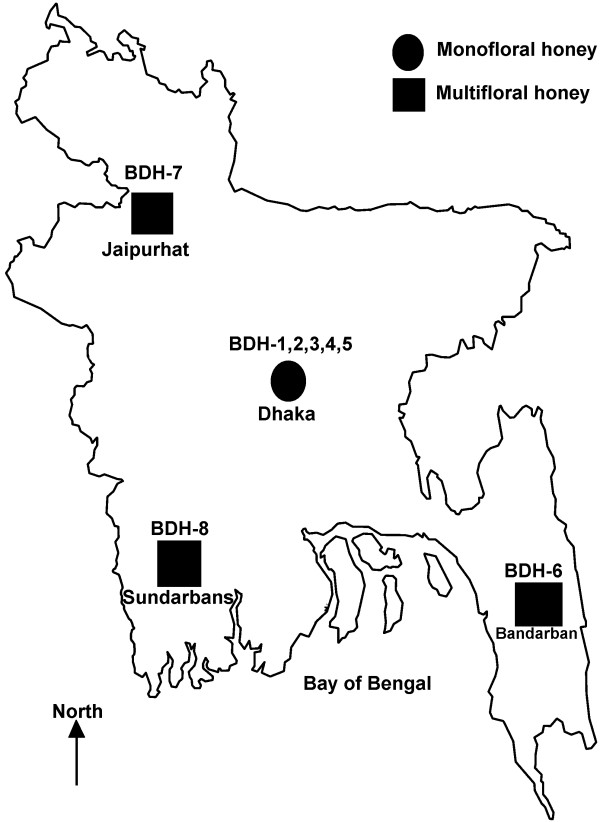
Location of honey sample collection points in Bangladesh.

Eight different honey samples were collected from different areas of Bangladesh between the months of June 2009 and August of 2009 and stored for 1.5 years from September 2009 to February 2011. Five of the samples were monofloral honey and three were multifloral, also known as “thousand flower honey”. *Apis dorsata* was the honeybee species for all the honey samples. All samples were stored at room temperature (20°-25°C) before analysis and were treated similarly (Table 1). All of the honey samples were investigated after 1.5 years of collection, which is the average shelf life of honey sold on the shelves. The geographical location of honey collection is presented in Figure 1.

**Table 1:** Details of eight different honey samples

**Name**	**Code name**	**Type**	**Scientific name of the plant source**
Bangladeshi honey-1	BDH-1	Monofloral	*Nigella sativa* (black seed or kali jeera)
Bangladeshi honey-2	BDH-2	Monofloral	*Sesamum indicum* (Til)
Bangladeshi honey-3	BDH-3	Monofloral	*Litchi chinensis* (Litchi)
Bangladeshi honey-4	BDH-4	Monofloral	*Guizotia abyssinica* (Ramtil)
Bangladeshi honey-5	BDH-5	Monofloral	*Brassica campestris* var. (Mustard)
Bangladeshi honey-6	BDH-6	Multifloral	Mixed source
Bangladeshi honey-7	BDH-7	Multifloral	Mixed source
Bangladeshi honey-8	BDH-8	Multifloral	Mixed source

### Physical analysis

#### pH

A 10% (w/v) solution of honey was prepared in milliQ water (Millipore Corporation, Massachusetts, USA) for pH measurement using a pH meter (Elico pH analyzer, Elico Pvt Ltd., Mumbai, India). The moisture content was determined using a refractometric method. In general, the refractive index increases with an increase in the solid content of a sample. The refractive indices of honey samples were measured at ambient temperature using an Atago handheld refractometer (KRUSS, HRH30, Germany), and the measurements were further corrected for a standard temperature of 20°C by adding the correction factor 0.00023/°C.

#### Moisture content

The moisture content was determined in triplicate, and the percentage of moisture content corresponding to the corrected refractive index was calculated using Wedmore’s table.

### Color analysis

The color intensity of the honey samples was measured according to the Pfund classifier. Briefly, homogeneous honey devoid of air bubbles was transferred into a cuvette with a 10-mm light path to about half the cuvette volume and was inserted into a Hanna HI 96785 color photometer (Hanna instruments, Cluj-Napoca, Romania). Color grades are expressed in millimeters (mm) Pfund grades when compared to an analytical grade glycerol standard reference. Triplicate measurements were performed for each sample by means of the United States Department of Agriculture (USDA) approved color standards [14].

### Electrical conductivity (EC) and total dissolved solids (TDS)

EC and TDS measurements were taken using conductivity meter (HI 98311, Hanna Instruments, Mauritius) for a 20% (w/v) solution of honey suspended in milliQ water [15]. The EC of the milliQ water was less than 10 μS/cm. The EC and TDS of each sample were analyzed in triplicate, and means were expressed in mS/cm and ppm, respectively.

### Color intensity: Absorbance at 450 nm (ABS_450_)

The mean absorbance of the honey samples was determined by the method of Beretta [16]. Briefly, the honey samples were diluted to 50% (w/v) with warm (45 - 50°C) milliQ water, and the solution was filtered using a 0.45-μm filter to eliminate large particles. The absorbance was measured using a spectrophotometer at 450 and 720 nm, and the difference in absorbance was expressed as mAU.

### 5-hydroxymethylfurfural (HMF) concentrations

The determination of HMF concentrations was conducted using a high-performance liquid chromatography (HPLC) method based on the method published by the International Honey Commission (IHC, 1999). Briefly, the honey samples (10 g each) were diluted to 50 ml with distilled water, filtered using a 0.45-μm nylon membrane filter and injected (20 μl) into an HPLC system (Waters 2695, Milford, MA, USA) equipped with a Photodiode Array Detector (Waters 2996). The HPLC column was a Merck Purospher Star, RP-18e, (125 × 4 mm, 5 μm) fitted with a guard cartridge packed with the same stationary phase (Merck, Germany). The HPLC method included an isocratic mobile phase, 90% water and 10% methanol with a flow rate of 1.0 ml/min. All solvents used were of HPLC grade. The detection wavelength was 200–450 nm with specific monitoring at 285 nm. The HMF concentration of each sample was calculated by comparing the corresponding peak areas of the sample and those of the standard solutions of HMF (Sigma-Aldrich, USA) after correcting for the honey dilution. There was a linear relationship (r^2^ = 0.9997) between the concentration and the area of the HMF peak (results are expressed in mg/kg).

### Biochemical analysis

#### Total sugar

The sucrose content of each honey sample was determined based on the refractometric method (Atago handheld refractometer, ATAGO, N-1α, Japan). Briefly, the honey samples were suspended in milliQ water to make a solution of 20% (w/v) concentration. The percentage of sucrose content was measured in g/mL honey.

#### Total phenolics

Phenolic compounds from honey samples were detected by a modified spectrophotometric Folin-Ciocalteu method [17]. Briefly, 1 mL honey solution (0.2 g/ml) was mixed with 1 mL Folin and Ciocalteu’s phenol reagent. After 3 min, 1 mL 10% Na_2_CO_3_ solution was added to the mixture and adjusted to 10 mL with distilled water. The reaction was kept in the dark for 90 min, after which the absorbance was read at 725 nm by a T 60 UV/VIS spectrophotometer (PG Instruments Ltd, UK). Gallic acid was used to calculate the standard curve (20, 40, 60, 80 and 100 μg/mL, r^2^ = 0.9970). The estimation of the amount of phenolic compounds was carried out in triplicate. The results were reported as the mean ± standard deviations and were expressed as mg of Gallic acid equivalents (GAEs) per kg honey.

#### Total flavonoids

The total flavonoid concentration of each honey sample was determined according to the colorimetric assay developed by Zhishen [18]. 1 mL honey solution (0.2 g/ml) was mixed with 4 mL distilled water. At baseline, 0.3 mL NaNO_2_ (5%, w/v) was added. After five min, 0.3 mL AlCl_3_ (10% w/v) was added, followed by the addition of 2 mL NaOH (1 M) six min later. The volume was immediately increased to 10 mL by the addition of 2.4 mL distilled water. The mixture was vigorously shaken to ensure adequate mixing, and the absorbance was read at 510 nm. A calibration curve was prepared by using a standard solution of Catechin (20, 40, 60, 80 and 100 μg/mL, r^2^ = 0.9880). The results were also expressed as mg Catechin equivalents (CEQ) per kg honey.

#### Total protein

The total protein content was determined by Lowry’s method [19] of protein estimation, which is based on the formation of a copper-protein complex and the reduction of phosphomolybdate and phosphotungstate present in Folin-Ciocalteau reagent to hetero polymolybdenum blue and tungsten blue, respectively. Bovine serum albumin (BSA) (0–100 μg/ml) was used as a standard for preparing the calibration curve.

#### Proline content

Proline content in the honey samples was measured using a method established by the IHC [19]. Briefly, BSA solutions were prepared by mixing stock BSA solution (1 mg/mL) to a final volume of 5 ml. The BSA concentration range was 0.05 to 1.00 mg/ mL. From the dilutions, 0.2 mL was transferred to different test tubes, and 2 mL of alkaline copper sulfate reagent (analytical reagent) was added before proper mixing. The solution was incubated at room temperature for 10 min. Then, 0.2 mL Folin Ciocalteau solution was added to each tube and incubated for 30 min. The absorbance was measured at 660 nm. The results were expressed as mg/kg.

### Ascorbic acid content

Determination of ascorbic acid content was done following the method described by Ferreira et al. [20]. Briefly, the sample (100 mg) was mixed with 10 ml 1% metaphosphoric acid for 45 min at room temperature and filtered through Whatman No. 4 filter paper. The filtrate (1 ml) was mixed with 9 ml 2,6-dichlorophenolindophenol (DCPIP) 0.005%, and the absorbance was measured within 30 min at 515 nm against a blank. The content of ascorbic acid was calculated on the basis of the calibration curve of authentic L-ascorbic acid (50, 100, 200 and 400 μg/ml; Y = 3.2453X - 0.0703; r^*2*^ = 0.9440), and the results were expressed as mg ascorbic acid/kg honey.

### Analysis of antioxidant activities

#### AEAC

The antioxidant content was determined by measuring AEAC values and was calculated using the method of Meda et al. [21]. Briefly, honey samples were dissolved in methanol to a final concentration of 0.03 g/ml. A 0.75-ml aliquot of the methanolic honey solution was then mixed with 1.50 ml 0.02 mg/ml DPPH solution prepared in methanol. The mixture was incubated at room temperature for 15 min, and the absorbance was measured at 517 nm using a spectrophotometer. The blank was 0.75 ml methanolic honey solution mixed with 1.5 ml methanol. Ascorbic acid standard solutions (1, 2, 4, 6 and 8 μg/ml) prepared in milliQ water were used to plot the calibration curve. Measurements were performed in triplicate, and the mean value was expressed as mg ascorbic acid equivalent antioxidant content per 100 g honey.

#### DPPH-free radical-scavenging activities

The antioxidant properties of each honey sample were also studied by evaluating the free radical-scavenging activity of the DPPH radical. The determination was based on the method proposed by Ferreira et al. [20]. Briefly, 0.8 mL honey solution (0.2 g/ml) was mixed with 2.7 mL methanolic solution containing DPPH radicals (0.024 mg/mL). The mixture was vigorously shaken and left to stand for 15 min in the dark (until their absorbances remained unchanged). The reduction of the DPPH radical was determined by measurement of the absorbance at 517 nm [22]. Butylated hydroxytoluene (BHT) was used as reference material. The radical-scavenging activity (RSA) was calculated as the percentage of DPPH discoloration using the equation % RSA = ([A_DPPH_– A_S_/A_DPPH_) × 100, where A_S_ is the absorbance of the solution when the sample solution has been added at a particular level, and A_DPPH_ is the absorbance of the DPPH solution.

### FRAP

The FRAP assay was performed according to a modified method described by Benzie & Strain [23]. Briefly, 200 μL properly diluted honey (0.1 g/mL) was mixed with 1.5 mL FRAP reagent. The reaction mixture was incubated at 37°C for 4 min, and then the absorbance was read at 593 nm against a blank that was prepared using distilled water. FRAP reagent was pre-warmed to 37°C and was freshly prepared by mixing 10 volumes of 300 mM/L acetate buffer (pH 3.6) with 1 volume of 10 mmol TPTZ solution in 40 mM/L HCl with 1 volume of 20 mM ferric chloride (FeCl_3_.6H_2_O). A calibration curve was prepared using an aqueous solution of ferrous sulfate (FeSO_4_.7H_2_O) at 100, 200, 400, 600 and 1000 μmol/L. FRAP values were expressed as micromoles of ferrous equivalent (μmol Fe [II]) per kg honey.

### Statistical analysis

The assays were carried out in triplicate, and the results were expressed as mean values and the standard deviation (SD). The statistical differences represented by letters were obtained through one-way analysis of variance (ANOVA) followed by Tukey’s honestly significant difference (HSD) post hoc test (p < 0.05). Correlations were established using Pearson’s correlation coefficient (r) in bivariate linear correlations (p < 0.01). These were carried out using Microsoft office Excel 2007 and SPSS version 16.0 program (IBM Corporation, New York, USA).

## Results and discussion

### Physical analysis

#### pH

Honey is characteristically acidic, with a pH ranging between 3.2 and 4.5. All of our honey samples were acidic, with pH values ranging between 3.6 and 4.1 (Table 2). These values are lower than those previously reported for honey samples from India [5], which have pH values between 3.7 and 4.4. Because it has been reported that low pH inhibits the presence and growth of microorganisms, Bangladeshi honey may have the potential to be used as good antibacterial agents. However, this needs to be further investigated.

**Table 2:** Physical characteristics of eight different Bangladeshi honey samples

**Sample**	**pH**	**Moisture (%)**	**EC mS/cm**	**TDS (ppm)**	**Color Intensity (mAU)**	**HMF (mg/kg)**
BDH-1	3.6 ± 0.0^e^	17.19 ± 0.1^g^	0.3 ± 0.001^e^	191.0 ± 0.0^f^	817.33 ±14.2^d^	11.96^c^
BDH-2	3.7 ± 0.0^d^	18.53 ± 0.1^e^	0.4 ± 0.001^d^	205.0 ± 1.7^e^	1217.67 ±11.6^c^	4.05^e^
BDH-3	3.9 ± 0.0^c^	19.19 ± 0.1^a^	0.2 ± 0.002^f^	104.3 ± 1.5^g^	254.00 ± 8.5^f^	3.18^g^
BDH-4	3.7 ± 0.0^d^	18.73 ± 0.1^c^	0.5 ± 0.001^c^	258.0 ± 1.0^d^	2021.67 ± 8.6^a^	43.81^b^
BDH-5	4.0 ± 0.0^b^	18.19 ± 0.1^f^	0.2 ± 0.002^f^	103.6 ± 0.5^g^	843.00 ± 16.6^d^	3.06^h^
BDH-6	4.1 ± 0.0^a^	18.66 ± 0.2^d^	0.7 ± 0.003^b^	496.3 ± 2.0^b^	785.33 ± 10.5^e^	703.10^a^
BDH-7	4.0 ± 0.0^b^	19.13 ± 0.1^b^	0.8 ± 0.001^a^	662.3 ± 1.1^a^	1738.33 ± 12.8^b^	6.59^d^
BDH-8	3.6 ± 0.0^e^	19.13 ± 0.1^b^	0.7 ± 0.002^b^	374.7 ± 1.5^c^	2034.00 ± 17.5^a^	3.87^f^

Data are expressed as mean ± SD. Significantly different values are represented by different letters (a, b, c, d, e, f, g, h) (p < 0.05).

#### Moisture content

The percentage of moisture content in the investigated samples ranged from 17.19 to 19.19, which are below 20, the maximum limit for the moisture content as per the Codex standard for honey [24]. The percent moisture content of Bangladeshi honey tends to be lower than other investigated honeys, such as 17.2-21.6% for samples from India [5] and 17.0-19.4% for samples from Turkey [25], despite the fact that the Bangladeshi honey samples had been stored for 1.5 years. As the moisture content present in honey samples is important and contributes to its ability to resist fermentation and granulation during storage, low moisture content in the Bangladeshi honey samples indicates its good storage ability.

#### Color analysis

**Figure 2:**
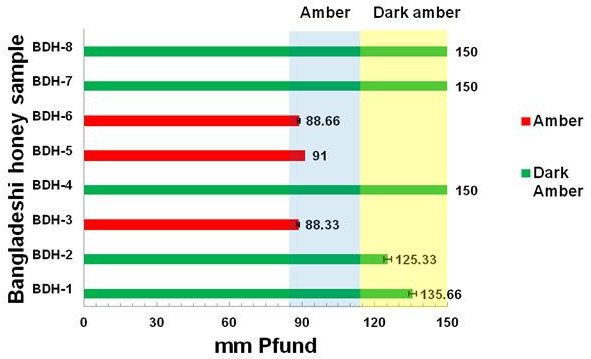
Color characteristics of Bangladeshi honey samples.

The color characteristics of the honey samples are presented in Figure 2. According to the USDA [14], honey samples with Pfund values less than 8 are classified as “water white”, between 9 and 17 mm are classified as “extra white”, between 18 and 34 mm as “white”, between 35 and 50 mm as “extra light amber”, between 51 and 85 mm as “light amber”, between 86 and 114 as “amber” and greater than 114 as dark amber. Based on this classification, Bangladesh honey can be classified as dark amber (for samples BDH-1, BDH-2, BDH-4, BDH-7 and BDH-8) or amber (BDH-3, BDH-5, BDH-6).

When all of the samples were compared, three of the samples (BDH-4, 7 and 8) had a very strong color at the highest Pfund value of 150 (Figure 2). Two of the samples (BDH-7 and 8) are multifloral honey, whereas BDH-4 is a monofloral honey from the plant *Guizotia abyssinica,* which has been reported to have high antioxidant properties [26].

#### Color intensity

The color intensity of the honey samples ranged from 254 to 2034 mAU (Table 2), which is comparable to those reported by other authors [5,6,27,28].

ABS_450_ is a reliable index for confirming the presence of pigments with antioxidant activities, such as carotenoids and some flavonoids. ABS_450_ was used because it has previously been reported that anthocyanins, a class of flavonoids present in functional drinks [27], and carotenoids have absorption maxima at 450 nm. In this study, we found that three dark amber samples (BDH-4, 7 and 8) also contained high levels of phenolic and flavonoid compounds. This indicates that color intensity may indicate higher phenolic and flavonoid concentrations or good antioxidant properties. Two of the samples (BDH-7 and 8) are multifloral honey, whereas BDH-4 is a monofloral honey including a plant that has been reported to have high antioxidant activities [26]. The correlation of color intensity with high levels of phenolics and flavonoids needs further investigation in future studies.

#### Total dissolved solids (TDS) and electrical conductivity (EC)

TDS is a measure of the combined content of the inorganic and organic substances contained in honey, including molecular, ionized, micro-granular (colloidal solution) or suspended forms. The TDS value of Bangladeshi honeys ranged between 103.6 and 662.3 (Table 2). TDS values are usually measured together with EC.

EC is an indicator of the botanical origin of honey. It has been reported that blossom honeys and mixtures of blossom and honeydew honeys ideally should have EC values of less than 0.8 mS/cm according to the European Union [29]. Similar to honey samples from India [5], the EC values of Bangladeshi honey samples varied between 0.2 and 0.8 (Table 2), which is within the range recommended by the European Union [29].

### HMF concentrations

HMF concentration is widely recognized as a parameter of honey freshness. This is because it is absent in fresh honeys, and its levels tend to increase during processing and/or due to aging. Several factors influence HMF levels, such as temperature, duration of heating process, storage conditions, pH and floral source; therefore, HMF levels provide an indication of overheating and storage in poor conditions [30].

Seven of the eight Bangladeshi honey samples had low HMF values (between 3.06 and 43.81 mg/kg) (Table 2). Contrary to our findings on Malaysian Tualang honey samples, in which HMF concentrations were found to be high (118.47–1139.95 mg/kg) when honey is stored for more than one year [30], HMF concentrations in Bangladeshi honey samples generally remained low despite the fact that the samples had been stored at room temperature (20°-25°C) for 1.5 years. The only exception to this was one sample (BDH-6) that had an HMF value of 703.10 mg/kg that exceeded the limits (80 mg/kg) established by European Community regulations [29]. It has been reported that fructose is unstable in acidic conditions, especially at pH 4.6. Because the rate of HMF formation in honey is dependent on pH and moisture content [30], we postulated that Bangladeshi honey samples with low pH levels (pH less than 4.6) and low moisture content may also contribute to the low HMF formation. This indicates that Bangladeshi honey may have unique characteristics that lead it to have good storage capability.

### Biochemical analysis

#### Total sugar

The total sugar content of the samples ranged from 42.8% to 60.6% (Table 3), which is similar to that of honey samples from India [5]. It is plausible that due to the acidity and low moisture content, sugars, especially glucose and fructose, were not converted to HMF even after storage at room temperature for 1.5 years; therefore, the sugar content remains high.

**Table 3:** Total levels of sugar, total phenolics, flavonoids, protein, proline, ascorbic acid, AEAC, DPPH and FRAP for the antioxidant capacity of eight different Bangladeshi honey samples

**Sample**	**Total sugar (%)**	**Phenolics (mg Gallic acid/kg)**	**Flavonoids (mg Catechin/kg)**	**Protein (mg/g)**	**Proline (mg/kg)**	**Ascorbic acid (mg/kg)**	**AEAC (mg/100g)**	**DPPH (% inhibition at mg/ml)**	**FRAP (μmol Fe (II)/100g)**
BDH-1	52.1 ± 0.4^c^	337.8 ± 4.6^d^	68.9 ± 0.5^d^	4.6 ± .01^e^	106.9 ± 0.5^h^	143.2 ± 2.2^c^	27.4 ± 1.7^d^	57.6^d^	247.8 ± 3.9^f^
BDH-2	55.3 ± 0.4^b^	648.6 ± 3.9^b^	143.0 ± 3.3^b^	7.5 ± .01^c^	681.7 ± 1.6^b^	135.5 ± 2.0^d^	25.4 ± 1.0^f^	59.8^d^	583.4 ± 9^d^
BDH-3	60.6 ± 0.9^a^	270.5 ± 2.3^e^	42.3 ± 2.2^g^	3.0 ± .01^g^	200.0 ± 1.0^g^	130.4 ± 1.7^g^	31.5 ± 1.5^c^	35.5^f^	140.2 ± 3.9^h^
BDH-4	42.8 ± 0.8^f^	641.2 ± 2.7^b^	126.0 ± 2.1^c^	8.1 ± 0^b^	2932.8 ± 3.7^a^	129.8 ± 3.4^h^	18.4 ± 0.7^h^	93.7^b^	617.8 ± 15.4^c^
BDH-5	55.3 ± 0.4^e^	265.0 ± 1.9^e^	48.0 ± 2.5^f^	3.5 ± .01^f^	512.8 ± 0.9^e^	154.3 ± 0.3^a^	32.3 ± 3.4^b^	50.0^e^	234.9 ± 15.4^g^
BDH-6	51.0 ± 0.9^b^	152.4 ± 5.3^f^	36.3 ± 3.0^h^	0.9 ± .02^h^	250.6 ± 2.1^f^	133.2 ± 2.5^f^	34.1 ± 1.4^a^	33.6^f^	260.4 ± 2.7^e^
BDH-7	51.0 ± 0.4^e^	473.9 ± 4.7^c^	61.9 ± 2.3^e^	8.6 ± .01^d^	581.9 ± 0.2^d^	134.7 ± 1.3^e^	22.2 ± 6.9^g^	74.5^c^	772.4 ± 2.5^a^
BDH-8	51.8 ± 0.4^d^	688.5 ± 5.9^a^	155.0 ± 6.9^a^	8.6 ± 0^a^	681.7 ± 0.4^c^	146.2 ± 2.8^b^	25.9 ± 0.2^e^	97.5^a^	663.9 ± 3.7^b^

Data are expressed as mean ± SD. In each column different letters indicate significant differences (p < 0.05).

### Total phenolics

The phenolic acid content determined using Gallic acid as standard was between 152.4 and 688.5 mg Gallic acid/kg (Table 3) (r^2^ = 0.995). The concentration and the type of polyphenolic substances in honey are variable and are reported to be dependent on the floral origin of the honey samples [25]. For example, Manuka honey from New Zealand was reported to have a total phenolic level of 434 mg Gallic acid/kg [28]. The mean total phenolic content in our samples (444 mg Gallic acid/kg) was slightly higher than Manuka honey, indicating that honey samples from Bangladesh are equally good if not superior in terms of antioxidant properties. Our results are also higher than that reported for honey samples from Cuba [31], Slovenia [6] and Burkina Faso [21]. The highest phenolic content was in sample BDH-8 (688.5 ± 5.9 mg Gallic acid/kg), which is a multifloral honey. It is possible that the blending of different variety of nectars from different flowers leads to a superior antioxidant properties in multifloral honey samples.

### Total flavonoids

All of the Bangladeshi honey samples had a lower content of flavonoids than polyphenols (mean = 72.15 mg Catechin/kg) (Table 3). The total flavonoid content in these samples were also slightly lower than that reported for Manuka honey (85.05 mg Catechin/kg) [28]. The range of Bangladeshi honey flavonoid concentrations was between 36 and 155 mg Catechin/kg, which is similar to that of Malaysian honey samples [28] but higher than that of samples from Cuba [31]. The highest flavonoid content was again found in sample BDH-8 (155 ± 6.9 mg Catechin/kg), a multifloral honey. It is possible that the blending of different variety of nectars from different flowers lead to more superior antioxidant property in multifloral honey samples, which should be further investigated.

### Protein

The protein content of Bangladeshi honey samples was between 0.9 ± .02 and 8.6 ± .01 mg/g (Table 3). Relatively higher protein levels ranging from 3.7 to 9.4 mg/g have also been reported in Algerian honey samples [4], whereas for honey samples from India, the content was reported to be lower (0.4 mg/g). The protein content can be attributed to the presence of different types of enzymes and other derived products that were introduced by the bees from the flower nectar. Protein levels in honey are dependent on the type of flora on which the bees forage [21].

### Proline

Published reports have revealed that various honeys contain between 11 and 21 different free amino acids, with proline being the main residue [32]. Proline is reported to mainly originate from bee salivary secretions during the conversion of nectar into honey. Protein levels are dependent on the type of flora that the bees visit and thus may be variable. Proline concentrations are an indicator of honey quality and of adulteration (suspected if proline levels are below 183 mg/kg) [33]. Except for a single sample (BDH-1, with a proline content of 106.9 mg/kg), all of our samples generally had high proline levels (above 183 mg/kg), indicating that the quality of the honey is good despite storage for 1.5 years.

The proline content in Indian honey samples has been reported to be between 133 and 674 mg/kg, whereas that in Algerian honey ranged between 202 and 680 mg/kg [4]. Four of our honey samples (BDH-2, 4, 7 and 8) showed high proline content and high phenolic and flavonoid content, which supports the findings from Meda [21], who reported that amino acid content in honey may contribute to its antioxidant activity.

### Ascorbic acid

In addition to polyphenols, honey contains a number of compounds known to act as antioxidants, including ascorbic acid and the enzymes glucose oxidase and catalase [34]. We detected moderate levels of ascorbic acid ranging from 129.8 to 154.3 mg/kg in our samples (Table 3), which is similar to previous studies on Portuguese honey (140 to 145 mg/kg) [20]. However, Malaysian Tualang honey samples were reported to have higher ascorbic acid content (140.6 to 360.0 mg/kg) [35] when compared to Bangladeshi honeys. Nevertheless, we cannot discount the fact that the ascorbic acid concentrations of our samples decreased with storage. It has been reported that when honey is stored for a long duration, the concentrations of several other compounds may also decrease, which can thus affect the levels of both ascorbic acid and enzymes.

### Analysis of antioxidant activities

#### AEAC

Using the standard curve of ascorbic acid (r^2^ = 0.9440), the AEAC values (mg AEAC/100 g of honey) of our honey samples ranged from 18.4 to 34.1 (Table 3). This is similar to that reported for the Burkina Faso honeys ranging from 10.20 to 37.87 mg AEAC/100 g [21] and Indian honey samples ranging from 15 to 30 mg AEAC/100g [5]. BDH-6, which is a multifloral honey, has the highest AEAC value, indicating high antioxidant potential.

#### DPPH

DPPH is a stable nitrogen-centered radical and has been widely used to test the free radical scavenging ability of various samples. The higher the DPPH scavenging activity, the higher is the antioxidant activity of the sample [5].

The percentage of DPPH scavenging activity of Bangladeshi honey ranged from 33.6% to 97.5% (Table 3), which is similar to honey samples from India (44%-71%) [5]. Four of the samples (BDH-2, 4, 7 and 8) had high DPPH inhibition activity (%), indicating that they have good antioxidant activities. These honey samples also have higher color intensity and are dark amber in nature. BDH-8, which is a multifloral honey, has the highest DPPH value, indicating high antioxidant potential. The second highest DPPH inhibition activity was shown by a monofloral honey from the plant *G. abyssinica,* which has been reported to have high antioxidant activities [26]. Interestingly, Ramadan et al. [26] reported that the plant *G. abyssinica* has higher antioxidant activity when compared to the plant *Nigella sativa;* accordingly, we found that BDH-4 honey sourced from *G. abyssinica* has higher antioxidant properties when compared to BDH-2 from *N. sativa*. Another multifloral honey (BDH-7) also had high antioxidant capacity.

#### FRAP

FRAP is a widely used method for antioxidant determination and has been used for the assessment of the antioxidant and reducing power of honey [7]. The FRAP assay gives a direct estimation of the antioxidants or reductants present in a sample based on its ability to reduce the Fe^3+^/Fe^2+^ couple.

The mean FRAP value of the honey samples was 440.15 mol Fe [II]/100g. The highest was 772.4 ± 2.5 μmol Fe (II)/100 g) (BDH-7, a multifloral honey), and the lowest was 140.23 μmol Fe [II]/100g (BDH-3, a monofloral honey) (Table 3), however the value is quite higher than the average monofloral honey value from Brazil which was 73.92 ± 7.50 [36]. Slovenian fir and forest honey had higher FRAP values of 478.5 μmol Fe [II]/100g and 426.4 μmol Fe [II]/100g, respectively. Alvarez-Suarez et al. [34] reported lower FRAP values (between 13.5 and 196.7 molM Fe [II]/100g) in Cuban honey samples, as did Beretta et al. [16] (72.8 to 1501.4 μmol Fe [II]/100g), indicating inferior antioxidant activities when compared to Bangladeshi honey samples.

### Correlation between antioxidant properties and biochemical parameters

Significant correlations exist between the biochemical and antioxidant parameters (Table 4). As established previously [31], the strongest positive significant correlation was found to be between total phenolics and total flavonoids. (r =0.9590) (Table 4). A strong positive correlation also exists between phenolics and FRAP (r = 0.8250), indicating that phenolics also contribute to the antioxidant capacity of honey. This statistically significant correlation was in agreement with the previous findings of, Saxena et al. [5], Kishore et al. [35] and Khalil et al. [37]. A significant correlation was also found between phenolics and color (r = 0.8200), as previously established [5,6,34], which indicates that color pigments may also be a strong indicator of the antioxidant properties of honey. The high correlation between phenols and DPPH activity (r = 0.869) shows that phenolic chemicals govern the antiradical potency, which was also proven by Beretta et al. [16]. Another significant correlation was found between flavonoids and FRAP (r = 0.691), as shown previously by Alvarez-Suarez [31] and Khalil [37]. A very high correlation between color and DPPH (r = 0.9480) and color and FRAP (r = 0.9140) indicates that there might be a strong correlation between color and the antioxidant capacity of honey, which was also described by Saxena et al. [5] and Kishore et al. [35]. In this study, proline showed moderate correlation with phenolics (r = 0.5690), flavonoids (r = 0.5060), DPPH (r = 0.6430) and FRAP (r = 0.4730), which indicates that there might be some contribution by proline in the antioxidant properties of Bangladeshi honey, as is the case in earlier studies in honeys of India [5] and Cuba [34]. Based on the results published by Meda [21], it can also be said that proline contributes to the antioxidant capacity of Bangladeshi honeys.

**Table 4:** **Correlation showing the interrelation among phenolics, flavonoids, DPPH, FRAP, ABS**_**450**_**and proline**

	**Phenolics**	**Flavonoids**	**DPPH**	**FRAP**	**ABS**_**450**_	**Proline**
Phenolics	1.000	0.959^**^	0.869^**^	0.825^**^	0.820^**^	0.569^**^
Flavonoids	0.959^**^	1.000	0.786^**^	0.691^**^	0.727^**^	0.506^*^
DPPH	0.869^**^	0.786^**^	1.000	0.820^**^	0.948^**^	0.643^**^
FRAP	0.825^**^	0.691^**^	0.820^**^	1.000	0.914^**^	0.473^*^
ABS _450_	0.820^**^	0.727^**^	0.948^**^	0.914^**^	1.000	0.647^**^
Proline	0.569^**^	0.506^*^	0.643^**^	0.473^*^	0.647^**^	1.000

** Correlation is significant at the 0.01 level (2-tailed).

* Correlation is significant at the 0.05 level (2-tailed).

## Conclusion

We studied several physicochemical and bioactive properties of monofloral and multifloral honey samples from different geographical areas of Bangladesh. Although the samples were stored for more than 1 year, they generally maintained their antioxidant properties, as indicated by the high phenolic and flavonoid contents. Bangladeshi honey samples also maintained the HMF concentrations despite being stored for 1.5 years, perhaps due to their acidic nature and low moisture content that prevent HMF formation. Both honey color and proline content are strong indicators for antioxidant capacity. In general, multifloral honeys have higher antioxidant properties based on their high levels of phenolics, flavonoids, AEAC, DPPH and FRAP values when compared to monofloral honeys. We also found that the honey samples from the monofloral honey sourced from *Guizotia abyssinica* and *Nigella sativa* had high antioxidant properties. Further studies of the antioxidant components of Bangladeshi honeys are required, especially the identification and quantification of individual flavonoids and phenolic acids.

## Abbreviations

DPPH: 1,1-diphenyl-2-picrylhydrazyl; TPTZ: 2,4,6-tris(1-pyridyl)-1,3,5-triazine; EC: Electrical Conductivity; TDS: Total Dissolved Solids; HMF: 5-hydroxymethylfurfural; HPLC: High Performance Liquid Chromatography; CEQ: Catechin Equivalents; DCPIP: 2,6-dichlorophenolindophenol; BHT: Butylated Hydroxy Toluene; FRAP: Ferric reducing/antioxidant power; ANOVA: Analysis of Variance.

## Competing interests

The authors declare that they have no competing interests.

## Authors’ contributions

MAI conceived and designed the study, undertook analysis and manuscript writing. MIK contributed to the design, conducted the study and contributed to the analysis of data. MNI conducted the study and contributed to the interpretation of data. MM helped to conduct the study. MAM collected the honey samples and contributed to the design of the study. SAS and GSH supervised the work, read and approved the final manuscript. All authors read and approved the final manuscript.

## Pre-publication history

The pre-publication history for this paper can be accessed here:

http://www.biomedcentral.com/1472-6882/12/177/prepub
